# Extract from an Article, Entitled Observations on Pericarditis

**Published:** 1856-10

**Authors:** Robert Law

**Affiliations:** Professor of the Institutes of Medicine in the School of Physic in Ireland


*Extract from an Article, entitled Observations on Pericarditis.* By ROBERT LAW, M. D., Professor of the Institutes of Medicine in the School of Physic in Ireland.

Have we any sign or signs, upon which we can rely as indicating adhesion of the pericardium?

Dr. Hope remarks: "I certainly consider this diagnosis to be one of the very few connected with the heart which cannot be made with absolute certainty, and I never, therefore, venture to assert respecting it."

40

He further remarks, that by a combination of signs he has succeeded in detecting it :—First, by the heart's beating as high up as natural in the chest, and causing a prominence of the cartilages of the left præcordial ribs. Second, and which he regards as the most characteristic of all, an abrupt jogging or tumbling motion of the heart, very perceptible in the præcordial region with the cylinder. Third, a history of previous pericarditis, especially if connected with acute rheumatism, affords strong presumptive evidence corroborating the preceding signs, and the absence of such history should make the auscultator pause before he ventures on a diagnosis in stronger terms than that it is probable or possible. With regard to the first sign stated by Dr. Hope, I would say, that I have never observed this high action of the heart and prominency of the cartilages of the left præcordial ribs in adhesion of the pericardium, but I have observed them in another morbid condition of the pericardium, in case of effusion into its cavity, when the fluid, gravitating toward the most dependent part, pushed up the organ towards its base, and produced a prominency corresponding to the base, the appearance which Louis designates "voussure." It is the pushed-up heart that forms the prominency, not the fluid, as is generclly believed.

As to the second sign proposed by Dr. Hope, I can only say, that his description of an abrupt, jogging, tumbling motion, does not describe the irregular action that I have sometimes observed in cases of adherent pericardium. Dr. Walshe's description of it as a tumultuous, confined action goes nearer to conveying an idea of what I have heard. As I have observed it, it has been a kind of pulling, dragging motion, as if one contraction of the ventricle was resolved into a series of short, abrupt contractions, sometimes so feebly and faintly expressed as if no impulse were communicated to the blood by the heart as it passed through it. The jogging, tumbling motion that I have observed has been in quite a different affection, viz., in the weak, gouty heart. M. Bouillaud remarks : "Je ne connois encore aucun signe qui puisse faire reconnaitre les adherences du pericarde." Dr. Sanders thought he had discovered a positive sign of an adherent pericardium in the retraction or dimple taking place during the systole of the ventricle in the epigastrium immediately below the false ribs, and which he ascribed to the diaphragm being drawn in by the ascending motion of the heart. Dr. Hope observes on Dr. Sanders' sign :—"I have searched for this attentively in several cases of adhesion, but have not been able to detect it in any degree that could constitute a sign. Dr. Heim, of Berlin, also proposed this sign. Laennec, speaking of it, says:—"J'ai cherchè inutilement depuis deux ans verifier cette observation chez tous les malades qui presentaient quelque signe de trouble de la circulation, et je n'ai jamais pu apercevoir le creux dont il s'agit, et dans le nombre de ces sujets il s'en est trouvè plusieurs dont le cœur adherait au pericarde." Dr. Stokes is the last authority to which I shall refer, as being the latest, and who had, therefore, the opportunity of testing the value of the signs of those who preceded him. He remarks :—"I more than doubt that there is any certain physical sign of adhesion of the pericardium, and have never been able to verify the sign of Dr. Hope of the double jogging impulse." I am bound to say, that none of the signs hitherto proposed has done more than to enable us to assert the likelihood of an adherent pericardium.

The sign which I propose, and with confidence, has this advantage, that we need not have followed the disease in its progress, nor do we require to have had any previous knowledge of the case. I have again and again tested its truth by post-mortem examination, as have others to whom I have communicated it, and have found that it may be relied upon. The sign to which I allude is, "*the persistence of the same extent of dulness to percussion in the præcordial region, no matter what position the individual may assume.*" The area of dulness on percussion in the præcordial region will be the same under every varying position of the body. The heart becomes so braced up that it cannot move as it does in its normal state, when, if examination be made, the patient either lying, or sitting, or standing, the results of percussion will vary accordingly, the dulness being greater in the first position, and less in the two latter. The individual himself, also, is quite conscious of the existence of some solid resisting body within his chest, which does not move in the changes of posture of his body, but impedes its motions.

I have proved this sign in cases where I have seen the patients all through their attack of pericarditis, and also in cases where the adhesion had been already formed, and have never found it to disappoint me. I, therefore, claim for it that it is *the physical sign* that may be relied on in proof of an adherent pericardium.—*Dublin Quarterly Journal of Medical Science.*

